# Clinical outcomes of DEB-TACE in locally advanced hepatocellular carcinoma: A 5-year real world experience

**DOI:** 10.1371/journal.pone.0309693

**Published:** 2024-09-12

**Authors:** Mina S. Makary, Jonathan Alexander, Luis E. Regalado, Sajid Jalil, Khalid Mumtaz

**Affiliations:** 1 Division of Vascular and Interventional Radiology, Department of Radiology, The Ohio State University Wexner Medical Center, Columbus, Ohio, United States of America; 2 Department of Radiology, Madigan Army Medical Center, Tacoma, Washington, United States of America; 3 Department of Radiology, Northwestern University Medical Center, Chicago, Illinois, United States of America; 4 Division of Gastroenterology, Hepatology, and Nutrition, Department of Internal Medicine, The Ohio State University Wexner Medical Center, Columbus, Ohio, United States of America; Hokkaido University: Hokkaido Daigaku, JAPAN

## Abstract

**Purpose:**

To evaluate outcomes including safety and efficacy of drug-eluting bead trans-arterial chemo-embolization (DEB-TACE) in the treatment of locally advanced hepatocellular carcinoma (LA-HCC).

**Materials and methods:**

In this single-center, retrospective study, we evaluated 471 consecutive patients with LA-HCC who underwent DEB-TACE from 2015 to 2020. Efficacy of DEB-TACE was assessed based on the imaging response using the modified Response Evaluation Criteria in Solid Tumors (mRECIST) and the biochemical response using alpha-fetoprotein (AFP) levels at 1-month follow-up. Adverse events, progression free survival (PFS), and overall survival were also examined.

**Results:**

HCC distribution was bilobar in 49% with largest lesion mean size of 4.3 cm ± 3.2, and a majority of patients (46.7%) were Barcelona Club Liver Cancer (BCLC) stage B. Complete radiologic response was achieved in 120 (25.5%) patients, comparable to a reported 28% rate for conventional TACE. Biochemically, 41 (8.7%) patients achieved complete response, and 113 (24%) had a partial response. A total of 59 (12.5%) patients were successfully bridged to liver transplantation. Major adverse events were observed in 3%, while 7.2% experienced post-embolization syndrome. Mean PFS was 6.7 months ± 6.6, and overall survival was 64%, 16.3%, 2.1% at 1, 3, and 5 years, respectively.

**Conclusion:**

Based on our real world experience at a single center, DEB-TACE remains the locoregional therapy of choice for LA-HCC due to its favorable safety and efficacy profile.

## Introduction

Hepatocellular carcinoma (HCC) represents the plurality of neoplastic liver masses with greater than 80% of non-metastatic liver lesions being diagnosed as HCC based on standard imaging and/or biopsy [1]. This translates to a large burden of cancer-related mortality attributable to HCC. In 2018, nearly 800,000 deaths worldwide were attributable to liver cancers, with roughly two-thirds of those affecting men [2]. Mortality mitigation for unresectable HCC is primarily achieved through screening, surveillance, staging, and selection of local and systemic therapies, with liver transplantation (LT) serving as a curative measure in selected cases and an accepted endpoint in treatment [3]. Standard of care for early stage HCC involves surgery–either resection, ablation, or transplant–intermediate stage HCC with loco-regional treatment, and at advanced stages includes systemic chemotherapy, immune check point inhibitors, or supportive care [4].

Due to a lack of appreciable symptoms in early stages and presence of portal hypertension in advanced liver disease, HCC therapy often precludes curative surgical intervention [5, 6]. This has driven a need for effective locoregional treatment (LRT) for disease control, downstaging, and bridging to resection or transplantation when feasible. These LRT include catheter-directed therapies encompassing transarterial radioembolization (TARE), transarterial chemoembolization (TACE), and transarterial embolization (TAE). TACE encompasses conventional TACE (cTACE) using an emulsified chemotherapy agent and drug-eluting bead TACE (DEB-TACE) which utilizes chemotherapy-coated beads. The comparable efficacy of DEB-TACE and cTACE has yet to reach a consensus, leading to institutions using either variant based largely on their preference. Recent analyses have determined that DEB-TACE results in lower systemic chemotherapy concentrations compared with cTACE; it also theoretically contributing to a more favorable adverse effect profile, while simultaneously achieving larger concentrations in cancer tissues [7]. Given lack of consensus on DEB-TACE efficacy compared to cTACE, particularly in real-world settings, this study aimed to address this gap by reviewing our single-center experience with DEB-TACE. The objectives of this study were to evaluate the efficacy of DEB-TACE in treating locally advanced HCC (LA-HCC), assess the adverse events associated with this treatment, and determine progression-free survival (PFS) and overall survival (OS) rates.

## Methods

### Study design and patient population

This retrospective cohort study was approved by the institutional review board (IRB). A total of 471 consecutive patients with LA-HCC were included. LA-HCC was defined as unresectable HCC including portal vein involvement without extrahepatic disease. Inclusion criteria were: patients aged 18 or older with a confirmed diagnosis of LA-HCC based on imaging and/or biopsy, deemed unsuitable for surgical resection or ablation per our multidisciplinary tumor board, and having undergone at least one session of DEB-TACE. Exclusion criteria included prior liver transplantation or participation in other concurrent clinical trials. All included patients underwent doxorubicin loaded DEB-TACE therapy at a single tertiary care medical center from December 2015 to December 2020. Data collection and analysis was performed between January 5^th^ and February 25^th^, 2021. Data was anonymized and reported in aggregate to protect patient’s privacy. Inclusion criteria for the study included a diagnosis of primary HCC confirmed by imaging criteria and/or pathological evaluation. Exclusion criteria included extra-hepatic disease, total Bilirubin (tBili) >3, and uncorrectable coagulopathy. Baseline characteristics of participants were obtained from the electronic medical record and are shown in Table 1.

**Table 1:** Baseline tumor characteristics and demographics of patients with hepatocellular carcinoma undergoing DEB-TACE.

Characteristic	N	±SD / Percent	Characteristic	N	SD/Percent
**Number of Patients**	**471**		**MELD Score**		
**Age (yrs)**	**64.58**	**±8.5**	<10	221	46.9%
**Gender**			10–19	214	45.4%
Male	373	79.2%	20–29	27	5.7%
Female	98	20.8%	**ALBI Grade**		
**Race**			1	93	19.8%
Caucasian	366	77.9%	2	313	66.6%
Black	77	16.4%	3	61	13.0%
Other/ Unknown	27	5.7%	**BCLC Classification**		
**Cirrhosis Etiology**			A	185	39.3%
Hep C	246	52.2%	B	220	46.7%
Hep B	26	5.5%	C	64	13.6%
ETOH	161	34.2%	D	0	0%
NASH	60	12.7%	**Past Liver Therapy**		
Cryptogenic	23	4.9%	None	227	48.2%
**ECOG Performance Status**			Resection	43	9.1%
’0	83	17.6%	Ablation	125	26.5%
’1	310	65.8%	TACE	160	34.0%
’2	71	15.1%	TAE	9	1.9%
’3	7	1.5%	TARE	21	4.5%
**Child Pugh Score**			Liver Transplant	3	0.6%
A	295	62.77%			
B	155	32.98%			
C	17	3.62%			

Baseline characteristics included patient demographic information (age, gender, and race) and etiology of cirrhosis) were examined. Disease severity was assessed utilizing various scores such as Model For End-Stage Liver Disease (MELD) score, Childs-Turcotte Pugh (CTP) stage, Barcelona Clinic Liver Cancer (BCLC) stage, and Albumin-Bilirubin grade. Patients’ performance status was assessed by using Eastern Cooperative Oncology Group (ECOG) score. History of prior liver therapy or interventions were further assessed. HCC burden was evaluated utilizing tumor size, location, vascular invasion, and extrahepatic disease involvement evaluated by computed tomography (CT) and/or magnetic resonance imaging (MRI).

### Procedural technique

Patients were admitted under hepatology inpatient service for procedure and monitored overnight for complications. DEB-TACE was performed under moderate sedation. Doxorubicin was used as chemotherapy for all patients and mean dose was 72.9 ± 27 mg. Patients routinely received prophylactic antibiotics, allopurinol, and hydrocortisone before each procedure. The procedural technique involved 5F femoral access and utilization of directional 5F catheters and microcatheters to select the appropriate arterial distribution based on tumor location under fluoroscopic guidance. DEB-TACE utilized 100 μm LC Beads (Boston Scientific Inc; Marlborough, MA) loaded with 100 mg of doxorubicin and mixed with 10 ml of iodinated contrast. Drug-eluting beads were administered under fluoroscopic monitoring until near stasis of flow–defined as the absence of anterograde flow–was achieved. This end point controlled the amount of chemotherapy delivered.

### Treatment response and analysis

Treatment response was assessed at the 1-month follow-up. Radiographic response was evaluated using the conditions proposed in modified Response Evaluation Criteria (mRECIST) [8]. Imaging modalities used were multiphasic contrast enhanced CT or MRI. Biochemical response was assessed utilizing alpha-fetoprotein (AFP) levels for secreting-tumors [9]. Clinical outcome included changes in liver function test, progression-free survival (PFS), and overall survival. DEB-TACE safety profile appraisal included peri- and post-procedural complications using the Society of Interventional Radiology (SIR) event classification [10]. The primary outcome were overall survival and complication rates. The secondary outcomes were PFS and response rates. Data extraction and analysis was performed using JMP 11 (SAS Institute Inc, Cary, NC).

## Results

### Periprocedural characteristics

Periprocedural characteristics including tumor burden, distribution, and DEB-TACE characteristics are summarized in Table 2. Hepatic disease distribution was 49% bilobar with largest lesion size of 4.3 ± 3.2 cm, and a plurality of patients exhibited a BCLC B classification (46.7%). 112 (25.9%) patients had an AFP > 8.5 and were categorized as AFP-secreting HCC. A repeat TACE in the same affected lobe was warranted for 157 (33%) patients after undergoing initial DEB-TACE. At one-month post-procedure, patients’ AST values demonstrated a trend towards improvement with a decrease of 5.4%, which approached but did not reach significance (P = 0.058). A similar improvement of 9.7% in ALT values was observed, while there was a significant increase in tBili of 15.4%, both of which reached statistical significance (P<0.01).

**Table 2:** DEB-TACE treatment details and periprocedural outcomes including adverse events stratified by SIR criteria.

Characteristic	N	SD/Percent
**Treatment Location**		
Bi-Lobar	10	2.1%
Lobar	299	63.5%
Segmental	162	34.4%
**Need for Additional TACE for Same Lesion**	157	33.3%
**Chemotherapy**		
Doxorubicin (mg)	72.9	±27.5
**Disease Burden**		
Solitary	90	19.1%
Multifocal	390	80.7%
**Disease Distribution**		
Unilobar	239	50.7%
Bilobar	232	49.3%
**Largest/Index Lesion Size**		
≤2 cm	105	22.1%
2–5 cm	235	49.9%
5–10 cm	94	20.0%
>10 cm	28	5.9%
**Tumor Mean Size (cm)**	4.3	±3.2
**Tumor Median Size (cm)**	3.3	
**Tumor Size Range (cm)**	0.7–17.8	
**Vascular Invasion**		
None	471	100%
**AFP Producing Tumor** *		
AFP >8.5 ng/ml	112	25.9%
**LFTs (Pre/Post)**		
AST (U/L)	64.3/60.8	-5.4% (P = 0.058)
ALT (U/L)	46.3/41.8	-9.7% (P<0.01)
Total Bilirubin (mg/dL)	1.3/1.5	15.4% (P<0.01)
**Length of Stay (Days)**	1.4	±2
**Minor Complications**		
***A***		
*None*		
***B***		
*Post-embolization Syndrome*	34	7.2%
**Major Complications**		
***C***		
*Liver Abscess*	1	0.2%
*Tumor Lysis Syndrome*	1	0.2%
*Acute Kidney Injury*	7	1.5%
***D***		
*Hematoma*	1	0.2%
***E***		
*Cardiac Event*	1	0.2%
*Liver Failure*	4	0.8%
***F***		
*None*		

*AFP producing tumor defined as abnormal serum lab value of >8.5 ng/ml.

### Adverse events and complications

The average length of stay of DEB-TACE patients was 1.4 days. Minor and major complications are outlined in Table 2. There were 34 minor complications in the form of post-embolization syndrome (7.2%). Major complications included three category C complications [(1 instance each of liver abscesses, tumor lysis syndrome, and 7 instances of AKI (1.9%)], one category D complication (1 hematoma), and two category E complications [(1 cardiac event and 4 acute liver failure (1%)]. These complications altogether represented 10% of our patients who underwent the DEB-TACE.

### Treatment response

Response to treatment according to mRECIST classification and biochemical responses is outlined in Table 3. Complete radiological response to DEB-TACE was achieved in 120 (25.5%) patients, while 206 (44.5%) had partial response at the 1-month follow-up. A total of 41 (8.7%) patients achieved normalization of AFP levels (complete response), and 113 (24%) had a partial improvement in AFP. Mean PFS was 6.7 months, and overall survival was 64%, 16.3%, 2.1% at 1, 3, and 5 years in patients undergoing DEB-TACE, respectively. A total of 59 (12.5%) patients received liver transplantation at some point following DEB-TACE.

**Table 3:** Post-DEB-TACE treatment outcomes including biochemical and radiographic responses as well as overall and progression-free survival.

Clinical Outcomes	N	SD/Percent
**Biochemical Response** *		
Complete Response	41	8.7%
Partial Response	113	24.0%
Stable Disease	133	28.2%
Progressive Disease	85	18.1%
**Radiographic Response (1-month)**		
Complete Response	120	25.5%
Partial Response	206	44.5%
Stable Disease	28	6.0%
Progressive Disease	85	18.1%
**Post-Procedure Liver Therapy**		
None	314	66.7%
Resection		
Ablation		
TACE	157	33.3%
TAE		
TARE		
Liver Transplant	59	12.5%
**Overall Survival**		
30-day	456	96.8%
1-Year	284	64.8%
3-Year	53	16.3%
5-Year	6	2.1%
**PFS (months)**	6.7	±6.6

*The biochemical response was assessed based on AFP levels. Levels decreased by at least 20% from pre-TACE baseline were considered partial response, and normalized levels considered complete response. A value of >8.5 ng/ml was considered normal.

## Discussion

HCC is a primary liver cancer with a huge morbidity and mortality burden worldwide. With the ultimate goal of bridging to liver transplant, many treatment options for various stages of disease have been stratified based on characteristics of the tumor and progression of disease as well as the functional status of the patient and their hepatic synthetic function [5, 6]. Between these, there has been a great deal of contention whether one subtype of embolization procedure is more effective than their counterparts. Malagari et al used a prospective model to compare DEB-TACE and TAE for HCC, finding that DEB-TACE had convincingly better local response and time free progression however they could not conclude any long-term survival benefit [11]. The ability of drug-eluting beads to deliver chemotherapy agents directly to target tissues should theoretically provide a significant benefit over bland embolization, and this presumably led to the greater local response after treatment and improved time to progression according to the authors. When comparing cTACE and DEB-TACE, Varela et al confirmed the efficacy of drug-eluting beads and once again showed their favorable pharmacokinetic distribution: systemic levels of doxorubicin were significantly lower in DEB-TACE treated patients [12].

Indeed, the initial response data we collected reflects the efficient way that DEB-TACE treats HCC lesions in the first few months. 25% of our patients had complete radiological response a month from treatment along with 8.7% achieving complete biochemical normalization–if it was elevated to begin with–while a further 44.5% and 24% had partial responses radiologically and biochemically respectively. In totality, nearly 70% of patients’ tumors had some degree of radiological responsiveness with 32.7% having a lower AFP as well. Work by Casadaban et al demonstrated a 28% objective response rate for cTACE [13]. Another similar study of patients receiving Y-90 TARE therapy, Salem et al conducted a prospective study of 291 patients resulting in between 42–57% total or partial response rate [14]. This indicates that, at least initially, DEB-TACE is a competitive treatment choice when compared with some of its contemporaries.

The long-term consequences of this initially promising immediate response are less clear. When repeated TAE and TACE were compared to conservative treatment by Llovet et al, they found that their 1-year survival for TAE was 75% while TACE 1-year survival was 82%; compared with 63% for their control group [15]. Our 1-year survival data was stratified by individual treatment session, not by patient, which likely lent to our lower 1-year survival of 64%. This finding is supported by the similar progression free survival (PFS) between the two studies; their group demonstrated a 6-month PFS in 35% of TACE patients while our average PFS was 6.7 months. A further study by Salem et al encompassing 100 patients over 15 years looked at long-term outcomes, breaking down average long-term survival by BCLC scale and finding survival of patients at stage A-C being 47, 25, and 15 months respectively [16]. Our average survival was not categorized by initial tumor class but was between 1 (68.3%) and 3 (16.3%) years which is consistent with their B stage average of roughly 2 years.

Regarding adverse events, a study by Lee et al in 2017, while confirming a preferable side effect profile, reported similar response rates between cTACE and DEB-TACE [17]. While suggesting that the two procedures may be similar in efficacy, the benefit of DEB-TACE may be in reaching higher levels of local chemotherapy agents for larger masses or in patients with impaired liver function without over-exposing the patient to systemically toxic doses. This is perhaps responsible for our favorable chemistry profile immediately post-procedure. While our patients’ liver function tests (LFTs) one month after embolization remained largely stable, Y90 embolization has demonstrated 11% grade 3 or 4 bilirubin toxicities post-procedurally [14]. Post-embolization syndrome was reported in 7.2% of patients, and other minor events accounted for only 38 more events. Major events were largely isolated with only single occurrences of most, the most recurrent of the remainder were 7 instances of AKI and 4 cases of liver failure. These results reflect an exceedingly low rate of adverse events. Post-embolization syndrome (PES)–a constellation of symptoms including pain, nausea, fatigue, fever, and increased LFTs–in particular is reported as being very common, some studies reporting an incidence of up to 60% [6]. While it is possible that our facility somehow evaded almost all PES, it is perhaps more likely that the retrospective nature of the study allowed for underreporting of adverse events, or it simply fell victim to the lack of standardization of terminology or location of reported events. Overall, the low incidence of serious adverse events indicates that DEB-TACE is a safe option to consider when deciding on embolization procedures to select based on aversion to poor immediate outcomes.

There are a few limitations in our study which need to be highlighted. First, survival and response were not broken down by disease class, which lumped our efficacy and survival data into a singular pool and did not distinguish between highly advanced and incidental cancers. Furthermore, the statistical analysis to elucidate efficacy by a difference in CP or BCLC classification would go far towards making our results more generalizable and understandable in terms of efficacy. Secondly, the retrospective nature of the study limited the availability of standardized reports regarding adverse events, outcomes, as well as standardized lab and radiological testing. The surprisingly low incidence of PES in particular suggests a lack of reporting that may have skewed our data. Additionally, post-treatment surveillance lab work and imaging after a month was not standardized. Future, prospective DEB-TACE studies should build upon these results by further standardization of reporting and testing.

In conclusion, this is a large single-center study that provides a comprehensive evaluation of DEB-TACE treatments. Our findings suggest that DEB-TACE treatment for locally advanced HCC is safe and efficacious. Initial treatment responses are encouraging as opposed to delayed results. However, average PFS is similar to other modalities. Future studies should continue to evaluate the practical differences between the types of embolization, especially in relation to initial tumor size and side effect profile.

## Abbreviations

HCC: Hepatocellular carcinoma
TARE: transarterial radioembolization
TACE: transarterial chemoembolization
TAE: transarterial embolization
cTACE: conventional TACE
DEB-TACE: drug-eluting bead TACE
IRB: institutional review board
LA-HCC: locally advanced hepatocellular carcinoma
MELD: Model For End-Stage Liver Disease
CTP: Childs-Turcotte Pugh
BCLC: Barcelona Clinic Liver Cancer
ECOG: Eastern Cooperative Oncology Group
CT: computed tomography
MRI: magnetic resonance imaging
mRECIST: modified Response Evaluation Criteria
AFP: alpha-fetoprotein

## References

[1] El-Serag HB, Rudolph KL. Hepatocellular carcinoma: epidemiology and molecular carcinogenesis. Gastroenterology. 2007 Jun;132(7):2557–76. doi: 10.1053/j.gastro.2007.04.061 17570226

[2] Ferlay J, Colombet M, Soerjomataram I, et al. Estimating the global cancer incidence and mortality in 2018: GLOBOCAN sources and methods. Int J Cancer. 2019 Apr 15;144(8):1941–1953. doi: 10.1002/ijc.31937 30350310

[3] Finotti M, Vitale A, Volk M, Cillo U. A 2020 update on liver transplant for hepatocellular carcinoma. *Expert Rev Gastroenterol Hepatol*. 2020;14(10):885–900. doi: 10.1080/17474124.2020.1791704 32662680

[4] Bruix J, Reig M, & Sherman M. Evidence-Based Diagnosis, Staging, and Treatment of Patients With Hepatocellular Carcinoma. Gastroenterology, 2016;150(4), 835–853. doi: 10.1053/j.gastro.2015.12.041 26795574

[5] Makary MS, Ramsell S, Miller E, Beal EW, Dowell JD. Hepatocellular carcinoma locoregional therapies: Outcomes and future horizons. *World J Gastroenterol*. 2021;27(43):7462–7479. doi: 10.3748/wjg.v27.i43.7462 34887643 PMC8613749

[6] Makary MS, Khandpur U, Cloyd JM, Mumtaz K, Dowell JD. Locoregional Therapy Approaches for Hepatocellular Carcinoma: Recent Advances and Management Strategies. *Cancers (Basel)*. 2020;12(7):1914. Published 2020 Jul 15. doi: 10.3390/cancers12071914 32679897 PMC7409274

[7] Song JE, & Kim DY. Conventional vs drug-eluting beads transarterial chemoembolization for hepatocellular carcinoma. World journal of hepatology, 2017;9(18), 808–814. doi: 10.4254/wjh.v9.i18.808 28706579 PMC5491403

[8] Lencioni R, Llovet JM. Modified RECIST (mRECIST) assessment for hepatocellular carcinoma. *Semin Liver Dis*. 2010;30(1):52–60. doi: 10.1055/s-0030-1247132 20175033 PMC12268942

[9] Bird TG, Dimitropoulou P, Turner RM, et al. Alpha-Fetoprotein Detection of Hepatocellular Carcinoma Leads to a Standardized Analysis of Dynamic AFP to Improve Screening Based Detection. *PLoS One*. 2016;11(6):e0156801. Published 2016 Jun 16. doi: 10.1371/journal.pone.0156801 27308823 PMC4911090

[10] Khalilzadeh O, Baerlocher MO, Shyn PB, et al. Proposal of a New Adverse Event Classification by the Society of Interventional Radiology Standards of Practice Committee [published correction appears in J Vasc Interv Radiol. 2018 Jan;29(1):146]. J Vasc Interv Radiol. 2017;28(10):1432–1437.e3. doi: 10.1016/j.jvir.2017.06.019 28757285

[11] Malagari K, Pomoni M, Kelekis A, et al. Prospective randomized comparison of chemoembolization with doxorubicin-eluting beads and bland embolization with BeadBlock for hepatocellular carcinoma. *Cardiovasc Intervent Radiol*. 2010;33(3):541–551. doi: 10.1007/s00270-009-9750-0 19937027

[12] Varela M, Real MI, Burrel M, et al. Chemoembolization of hepatocellular carcinoma with drug eluting beads: efficacy and doxorubicin pharmacokinetics. *J Hepatol*. 2007;46(3):474–481. doi: 10.1016/j.jhep.2006.10.020 17239480

[13] Casadaban LC, Minocha J, Bui JT, et al. Conventional Ethiodized Oil Transarterial Chemoembolization for Treatment of Hepatocellular Carcinoma: Contemporary Single-Center Review of Clinical Outcomes. AJR Am J Roentgenol. 2016 Mar;206(3):645–54. doi: 10.2214/AJR.15.14758 26901023

[14] Salem R, Lewandowski RJ, Mulcahy MF, et al. Radioembolization for hepatocellular carcinoma using Yttrium-90 microspheres: a comprehensive report of long-term outcomes. Gastroenterology. 2010;138(1):52–64. doi: 10.1053/j.gastro.2009.09.006 19766639

[15] Llovet JM, Real MI, Montaña X, et al. Arterial embolisation or chemoembolisation versus symptomatic treatment in patients with unresectable hepatocellular carcinoma: a randomised controlled trial. *Lancet*. 2002;359(9319):1734–1739. doi: 10.1016/S0140-6736(02)08649-X 12049862

[16] Salem R, Gabr A, Riaz A, et al. Institutional decision to adopt Y90 as primary treatment for hepatocellular carcinoma informed by a 1,000-patient 15-year experience. Hepatology. 2018;68(4):1429–1440. doi: 10.1002/hep.29691 29194711

[17] Lee YK, Jung KS, Kim DY, et al. Conventional versus drug-eluting beads chemoembolization for hepatocellular carcinoma: Emphasis on the impact of tumor size. *J Gastroenterol Hepatol*. 2017;32(2):487–496. doi: 10.1111/jgh.13501 27503585

